# A Differential Confocal Sensor for Simultaneous Position and Slope Acquisitions Based on a Zero-Crossing Prediction Algorithm

**DOI:** 10.3390/s23031453

**Published:** 2023-01-28

**Authors:** Tingyu Wang, Zhiyi Wang, Yongqiang Yang, Xiaotao Mi, Yunzan Ti, Jianli Wang

**Affiliations:** 1 Changchun Institute of Optics, Fine Mechanics and Physics, Chinese Academy of Sciences, Changchun 130033, China; 2 College of Materials Science and Opto-Electronic Technology, University of Chinese Academy of Sciences, Beijing 100049, China; 3 Jilin Provincial Key Laboratory of Intelligent Wavefront Sensing and Control, Changchun 130033, China

**Keywords:** confocal microscopy, real-time optical imaging, position sensor, slope sensor, zero-crossing prediction algorithm, surface prediction

## Abstract

A new sensor type is proposed to accurately detect the surface profiles of three-dimensional (3D) free-form surfaces. This sensor is based on the single-exposure, zero-crossing method and is used to measure position and angle simultaneously. First, the field intensity distribution in the posterior focal plane of the confocal microscope’s objective was modeled accurately. Second, because the camera needs to trigger acquisition when the surface (to be measured) reaches the focal position of the sensor, a zero-crossing prediction method based on a sliding window was proposed. Third, a fast, spatially convergent, peak-extraction algorithm was proposed to improve the accuracy and efficiency of peak extraction. This scheme reduces system installation and adjustment difficulties, and the single-exposure, zero-crossing method achieves high-speed, real-time image acquisitions. The experimental results indicate that the average error of the zero-crossing prediction system was 17.63 nm, the average error of the tilt degree measurement was 0.011° in the range of 0–8°, and the prediction error of the tilt direction measurement was 0.089° in the range of 0–360°. The sensor can measure the slope and can be potentially used for 3D surface precision detection.

## 1. Introduction

Optical free-form surfaces represent a new optical technology type. Owing to its superior surface freedom and powerful aberration balance capability [1], it has been extensively applied in many fields, such as remote sensing [1], transportation [2], and biosensing technology [3]. Free-form surfaces generally exhibit asymmetric and irregularly shaped characteristics [4]. Tremendous progress has been achieved in recent decades in the design and machining of aspheric surfaces. However, the development of free-form surface design, machining, and testing is relatively slow, and the detection technology of free-form surfaces has become the most important aspect responsible for limited applicability in the field of precision optics.

Compared with interferometry, the profiler does not require a compensating mirror system because of its measurement versatility. Compared with coordinate measuring machine (CMM), the noncontact optical probe avoids the risk of scratching the surface and has gained extensive attention in the field of free-form surface measurements [5]. The profiler uses a probe-scanning method to directly test the profile of the measured surface to obtain the three-dimensional (3D) profile information of each sampling point [6]; it then obtains the profile error through analysis, fitting, and reconstruction. Differential confocal microscopy is an ideal noncontact optical probe that has absolute measurement and focusing tracking advantages and can improve the focusing sensitivity, sensor linearity, and signal-to-noise ratio (SNR) responses. Most importantly, differential confocal microscopy increases the resolution in the axial direction. The laser differential confocal microscope (DCM) uses the linear region of the axial response near the zero point to obtain the axial position of the current real-time point measurement by solving the problem of finding the position of zero-crossing [7,8,9].

In the process of data acquisition, a noncontact probe is spatially shifted by a multidimensional motion mechanism during sampling. Owing to moving mechanism errors, the obtained point-cloud array is neither regular nor uniform. In the field of surface reconstruction, some published studies have proven that slope measurements enhance the detection ability compared with 3D coordinate measurements. Khairi et al. [10] proposed a method to reconstruct mirror surface shapes using normal vectors. They used a 5-degree-of-freedom (DOF) camera system to extract the normal vector of a curved surface. These normal vectors were then used as data for the cubic polynomial functions to reconstruct the shape of the surface. The experimental results showed that this method can improve the accuracy of 3D shape measurements. At the same time, slopes (rather than 3D coordinates) are used in the surface shape-detection process [11]. The latter process is associated with detection errors induced when the curvature of the surface is much higher than that of the defect-free area. However, these techniques have significant defects in the fuzzy surface reconstruction of gradient fields. Although the problem of non-integrability of the gradient field can be solved by regularization, frequency domain integration, or least-squares fitting techniques, there are still reconstruction limitations. Additionally, the measurement method that only obtains the slope usually has problems (attributed to large cumulative errors) and the coupled cumulative error among the measured points cannot be decomposed. Pan et al. [12] used spatial locations and slopes to reconstruct complex surfaces and proved the effectiveness and robustness of the proposed method. In addition, obtaining the spatial position and spatial slope simultaneously can dynamically optimize the sampling strategy and assist path planning.

In previous studies, the detection process of a confocal system was regarded as an ideal point contact, and the optical axis was required to be perpendicular to the measured surface. Some studies have proven that the local tilt angle of the measured surface can induce measurement errors in confocal microscopy systems. Mauch et al. [13] explained in detail the signal formation process of the confocal system and proved that when the measured surface is curved, the defocusing wavefront may have a larger coincidence ratio than the focusing wavefront and the curvature of the measured surface, thus resulting in a large deviation in the axial position corresponding to the extreme value of the confocal signal strength. Beguelin et al. [14] used machine-learning methods to compensate for errors caused by surface tilts in distance measurements and used imaging results to correct the measured data. Therefore, it is also important for the measurement of the spatial position to obtain the surface inclination while 3D spatial coordinates are concurrently obtained.

Therefore, the system used to obtain simultaneously the spatial position and slope has gradually become a research focus owing to the aforementioned advantages. However, few methods that can measure both position and tilt are known in the literature. These systems and measurement methods are associated with many problems, such as complex system structures and miscellaneous data processing, and their accuracy characteristics cannot meet practical application needs [15,16,17,18]. Wang et al. [15] added a pair of spatially orthogonal, double-cylinder mirrors combined with a linear charged-coupled device (CCD) structure to the traditional DCM system, and successfully achieved dimensional reduction; this transformed the problem of two-dimensional (2D) peak search to a peak-extraction problem of one-dimensional data, and thus achieved high measurement accuracy. The mean prediction errors in the 2D plane tilt angle from −10° to +10° were 0.0134° (0.067% full-scale (F.S)) and 0.0142° (0.071% F.S). At the same time, this structure effectively utilizes the high-speed response characteristic of a linear CCD and can satisfy the basic principle of high-speed scanning of the DCM. Although this type of structure can achieve a higher measurement accuracy and a larger measurement range, the sensor is limited owing to the high-installation accuracy and stringent system parameter requirements. First, regardless of the axial or radial direction, once the placement of the linear CCD is offset, the effective information cannot be fed into the system, thus resulting in the loss of effective signals, or may even lead to the inability to obtain signals. Second, if the linear CCD is not accurately placed on the focal plane of the cylindrical mirror, the SNR is reduced; this affects the peak-extraction results and reduces the measurement accuracy. Finally, the system needs to satisfy a relatively strict system parameter design related to the numerical aperture (NA) of the cylindrical mirror and pixel size of the linear CCD; these requirements limit its application.

In this study, we propose a zero-crossing prediction algorithm based on the sliding-window concept. The algorithm achieves accurate single-frame acquisition, thus successfully overcoming the limitations of the DCM system owing to the impact of exposure time, frame-readout time, and data-transmission bandwidth during acquisitions in the continuous image mode [19]. The proposed algorithm was verified experimentally. The error was much smaller than the peak-extraction error and was ignored. In addition, a fast, spatially converging, 2D peak-extraction algorithm was proposed, and the effectiveness and feasibility of the algorithm were verified by simulations and experiments from the perspectives of precision, speed, and peak-extraction robustness. 

This study is structured as follows: Section 2 describes the optical path structure and mathematical model of the area scanned by a camera that received signals when the measured surface was tilted. The zero-crossing prediction algorithm based on the sliding-window concept is introduced in Section 3. In Section 4, the principle and flow of a fast, spatially converging, 2D peak-extraction algorithm are introduced. Simulation verifications are presented in Section 5. In Section 6, we present the detailed physical experimental results and discuss the remaining problems and limitations of this study. Finally, the conclusions of this study are outlined in Section 7. 

## 2. Numerical Model and System Design

**Figure 1:**
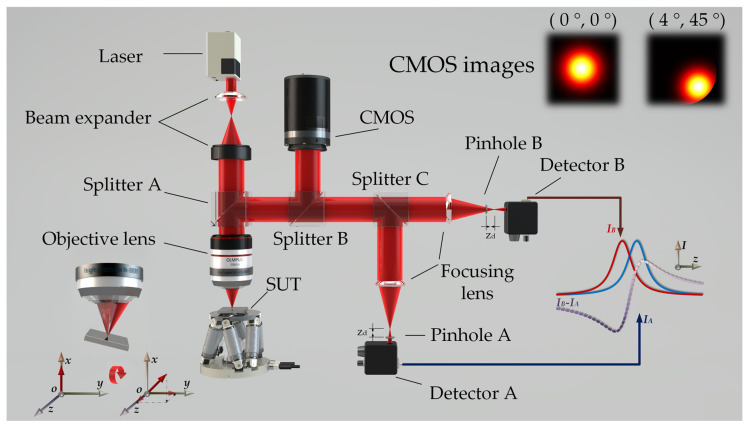
Diagram of the slope measurement sensor based on the differential confocal method showing the intensity distribution of the return beam spot detected by the detector when the measured plane is tilted.

The structure of the slope-measurement sensor based on differential confocal microscopy is shown in Figure 1. After beam expansion, the parallel beam emitted from the laser enters the microscope’s objective lens and is focused on the surface under test (SUT). The light beam reflected by the measured component is collected by the objective lens, reflected by the beam splitter mirror A, and then divided into the differential ranging and a tilt-measurement beams by the beam splitting mirror B. Differential ranging structures use beam splitter C to divide the beam into two paths. After the two beams are focused by the focusing lens, the energy of the beam is measured by the two prefocus and postfocus pinhole detectors, which are at the same distance away from the focal plane of the focusing lens. With this structure, the focal position of the sensor’s objective can be accurately located [20]. When the measured surface is on the focal plane of the microscope’s objective lens, the slope measuring unit in the sensor analyzes and processes the returned light field to obtain the tilt angle corresponding to the current spatial position of the SUT accurately.

**Figure 2:**
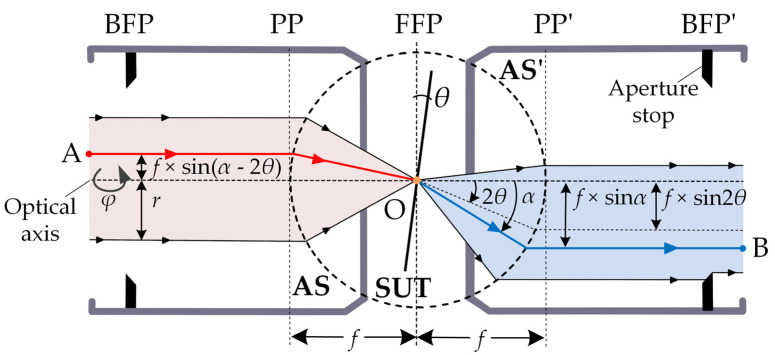
Schematic of ray tracing when the structure of the microscope’s objective with a high NA meets the sine condition of Abbe is expanded to measure the inclination plane. The left side of the SUT is the incident light path, and the right side of SUT is the reflected light path.

For a high-magnification objective with a large numerical aperture (NA), the aberration should be fully corrected to ensure excellent imaging quality. In this case, the optical path must satisfy the Abbe sine condition [21]. Because most commercial microscope objectives satisfy Abbe’s sine condition [22], we used the aplanatic lens model that has been used in similar research. Figure 2 shows an expansion model of the microscope’s objective lens, where BFP is the back focal plane, PP is the principal plane of the microscope, AS is the Abbe sphere, and FFP is the front focal plane. In an objective lens with a high thickness, the collimated beam is incident from the BFP and forms a spherical wave (with a focal length radius *f*) centered on the focal spot.

When the objective satisfies the Abbe sine condition, the plane wave passes through it and is converted into a convergent spherical wave. The spherical wave converges on the SUT at the focal point and re-enters the objective lens after it is reflected by the SUT. The position at which the beam re-enters the objective lens depends on the tilt angle of the measured surface. The rays reflected at the focal point *O* and at an angle *α* with respect to the central axis of the objective lens will be intercepted by AS’ at a distance *f* × sinα from the axis; they will then enter PP’ along the direction parallel to the central axis of the objective lens and emitted from the BFP’. As the aperture stop is usually placed on the BFP’, some beams reflected by the SUT cannot re-enter the optical path owing to the interception of the aperture stop when the inclination of the SUT is increased.

When the 2D tilt angle of the SUT is (*θ*, *φ*), the included angle between the normal unit vector $\vec{n}$ of the SUT and the central axis of the microscope is *θ*, and the azimuthal angle is *φ*, we refer to *θ* as the tilt degree and *φ* as the tilt direction, as shown in Equation (1). $n_{x}$, $n_{y}$, and $n_{z}$ are the components of $\vec{n}$ in the rectangular coordinate system.

(1) $$\vec{n}=(n_{x},n_{y},n_{z})=(\sin\theta \;\times \;\cos\varphi ,\;\sin\theta \;\times \;\sin\varphi ,\;\cos\theta )$$

The process of incident light from the AS to the focal position, its reflection from the SUT at the focal position, and re-entry to AS’, can be considered as the incident light beam at the focal position; light is then rotated by 180° around the normal unit vector $\vec{n}$ of the SUT and is returned to the objective. The focus is on the origin *O*(0, 0, 0). The beam incident from position ${A(x}_{A},y_{A},z_{A})$, after its reflection by SUT, exits from position ${B\;(x}_{B},y_{B},z_{B})$. Using the axis vector rotation rule about axes [23], we can obtain the following spatial relationship [see Equation (2)] between positions *A* and *B*,

(2) $$\left[\begin{array}{c} x_{A}\\ y_{A} \end{array}\right]=-\left[\begin{array}{c} x_{B}\\ y_{B} \end{array}\right]+\left[\begin{array}{c} n_{x}\\ n_{y} \end{array}\right]\left[\begin{array}{ccc} n_{x} & n_{y} & n_{z} \end{array}\right]\left[\begin{array}{c} x_{B}\\ y_{B}\\ \sqrt{f^{\;2}-x_{B}{}^{2}-y_{B}{}^{2}} \end{array}\right]\times 2,$$

The light intensity at point *A* in the incident light direction was $I_{A}$, and the light intensity at point *B* in the reflected light direction was $I_{B}$. On the sphere of Abbe, the areas of the incident beam at point *A* and the outgoing beam at point *B* projected onto the sphere along the central axis of the microscope are different, thus resulting in the light field intensity inconsistency at points *A* and *B*. In the case of the total reflection of the incident light on the SUT, the energies of the incident and reflected lights are equal, and the light intensity is inversely proportional to the cross-section area of the lights. Therefore, by calculating the area of the light projected along the central axis of the microscope on the Abbe sphere, the light intensity $I_{B}$ at point *B* can be calculated using Equation (3) as follows,

(3) $$I_{B}{=I}_{A}\times \sqrt{\frac{\;f^{\;2}-x_{A}{}^{2}-y_{A}{}^{2}}{f^{\;2}-x_{B}{}^{2}-y_{B}{}^{2}}},$$

Therefore, when the field intensity distribution of the incident light on the BFP is known and the 2D tilt angle of the plane (to be measured) is (*θ*, *φ*), the field intensity distribution of the outgoing light on the BFP’ can be obtained using the position correspondence between the incident beam and the outgoing beam (Equation (2)) and the light intensity conversion relation (Equation (3)).

The radius of the parallel Gaussian beam entering the pupil of the microscopic objective is *r*; this represents the distance from the center at the position where the energy drops by 1e2 with respect to that at the center brightness. When the SUT does not tilt, the peak position of the reflected beam’s field intensity distribution on BFP’ is located at the center of BFP’. When the SUT tilts at different angles, the peak position of the field intensity distribution of the reflected beam on BFP’ will be offset at different values; the offset of the peak position corresponds to the tilt angle of the SUT, as shown in the upper right corner of Figure 1.

In a recent study, Wang [15] scanned a surface with an objective lens, used a data board card to establish the corresponding relationship between the real-time image information received by the two linear CCDs orthogonal in space and the axial position of the scan, and then transmitted it to the computer for storage. After the signal was calculated, the zero-crossing position was obtained, and the information related to the current measured point was extracted based on the established relationship. At this point, the selected signal relied on the excellent axial tomographic capability of the DCM to locate the focal location accurately. Therefore, the image information obtained at focus was considered to be the image information selected by the linear CCD. Although the scanning camera can avoid installation and adjustment problems, there are limitations in its application; when this camera operates in the continuous image acquisition mode, it is affected by the exposure time, frame-readout time, and data-transmission bandwidth. Accordingly, the frame rate can only reach hundreds of Hz, which is not suitable for high-speed, continuous image acquisition. However, when the scanning camera operates in the single-frame image acquisition mode, it is not affected by the single-frame image readout time and data-transmission bandwidth and can complete the acquisition of a single image within at least a few seconds; this is suitable for high-speed capturing of moving objects. Therefore, if the existing scanning data can be used to predict the focal depth range accurately and the focal plane location during the scanning process, the scanning camera can be set to operate when it is focused. This means that each scanning period corresponds to a zero-crossing-image returned from the scanning camera; the current slope can then be estimated.

## 3. Zero-Crossing Prediction Algorithm Based on Sliding-Window Concept

The prediction algorithm based on sliding window has been widely used in trajectory prediction [24] and real-time prediction [25]. To solve the prediction problem of the zero-crossing time of the differential signal, a prediction algorithm for the zero-crossing time based on a sliding window is proposed. At the zero-crossing position of the differential signal curve, a scanning camera was used to collect the frame image and measure the 2D tilt angle of the tested position. For a perfect, coherent imaging, aberration-free optical system, as shown in Figure 1, the differential responses of the prefocal pinhole detector A and postfocal pinhole detector B are expressed by Equation (4) [26],

(4) $$I_{D}\left(z\right)=\exp\left(\frac{{-2D}_{ph}{}^{2}}{{\left(\frac{{2f}_{2}\lambda }{\pi r}\right)}^{2}\left(1+{\left(\frac{{\pi r}^{2}z_{d}}{f_{2}{}^{2}\lambda }+\frac{{2\pi r}^{2}z}{f_{1}{}^{2}\lambda }\right)}^{2}\right)}\right)-\exp\left(\frac{{-2D}_{ph}{}^{2}}{{\left(\frac{{2f}_{2}\lambda }{\pi r}\right)}^{2}\left(1+{\left(\frac{{-\pi r}^{2}z_{d}}{f_{2}{}^{2}\lambda }+\frac{{2\pi r}^{2}z}{f_{1}{}^{2}\lambda }\right)}^{2}\right)}\right)$$

where *z* is the displacement of the SUT relative to the focal plane of the objective, *λ* is the wavelength of the laser beam, $f_{1}$ is the focal length of the objective, $f_{2}$ is the focal length of the imaging lens, *r* is the radius of the Gaussian beam incident on the microscopic objective, $z_{d}$ is the offset of the image square hole from the focal plane of the condensing lens, and $D_{ph}$ is the pinhole diameter. By analyzing Equation (4), the differential confocal signal has a unique absolute zero when the signal strengths of the two pinhole sensors are equal. The zero-crossing of the differential signal corresponds to the SUT at the focal point of the microscope.

The required exposure time is $T_{1}$ when the camera performs single-frame image acquisition. At the zero-crossing time $t_{0}$, the SUT is at the focal position of the microscope. To obtain the field intensity distribution of BFP’ when the SUT is at the focal point of the objective lens, the camera needs to initiate the exposure at $t_{0}-{\;T}_{1}/2$ and terminate it at $t_{0}{+T}_{1}/2$. In the actual operation process, the control board needs to predict the arrival time $t_{0}$ at least $T_{1}/2$ μs in advance according to the data of the two pinhole detectors, and trigger camera acquisitions (exposures) at the time $t_{0}-{\;T}_{1}/2$ to initiate the spot image acquisitions.

**Figure 3:**
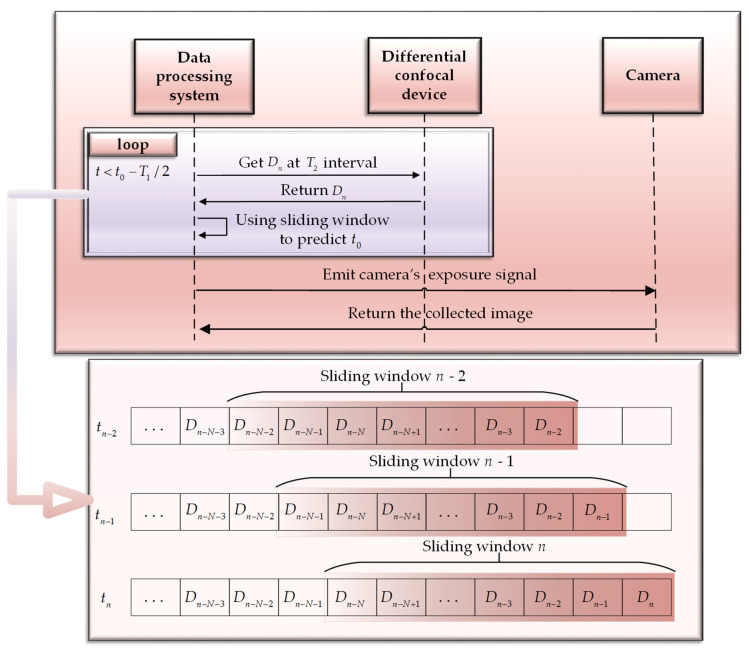
Schematic depicting the sliding-window concept. Data in the window are automatically updated as time progresses, and they are assigned different weights according to the order of collection.

During the scanning process near the focal point, motion of this sensor can be approximated as uniform along the axial direction. The data acquisition board card collects a set of differential signals at $T_{2}$ intervals. As shown in Figure 3, at time $t_{n}$, a group of differential signals $D_{n}$ is collected, and $D_{n}$ in the sliding window and its previous *N* groups of differential signals {$D_{n-N}$, …,$\;D_{n}$} are extracted. The workflow of the prediction of the zero-crossing time based on the sliding window concept is presented in Algorithm 1.
**Algorithm 1.** Zero-crossing time prediction algorithm based on the sliding window concept**Input:** Camera exposure time: $T_{1}$
    Differential signal sampling interval: $T_{2}$
    Differential signal data flow: $D_{n}$
**Output:** Camera on exposure signal 1: Initialize size of sliding window: *N*←${10\;\times \;T}_{1}{/T}_{2}$
2: Repeat 3: Collect a group of differential data $D_{n}$ at $T_{2}$ ns interval 4: Use the sliding window to extract data $D_{slider}$:$\left\{D_{n-N},D_{n-N+1},\;\ldots ,D_{n}\right\}$
5: Assign different weights to the data in the sliding window $D_{slider}$ in chronological order: D~i=e−(i−n)22N2×Di
6: Use the data in $D_{slider}$ to establish the prediction equation: D=k × t+b
7: Use the prediction equation to predict the zero-crossing time: $t_{0}=-b/k$
8: Until: current time $t\;\geq {\;t}_{0}-T_{1}/2$
9: Emit camera’s exposure signal

As shown in Figure 3, the data in the sliding window were assigned different weights according to the time sequence (as shown in Algorithm 1, step 4), and the prediction equation between time *t* and differential signal *D* was established, *D* = *k* × *t* + *b*, to minimize Equation (5),

(5) $$\sum_{i=n-N}^{n}e^{-\frac{{\left(i-n\right)}^{2}}{{2N}^{2}}}{\left(D_{i}-{k\times t}_{i}-b\right)}^{2},$$

Using the least squares rule [27], let the partial derivative of Equation (5) with respect to *k* and *b* be zero. Then, establish the system of equations, as shown in Equation (6),

(6) $$\{\begin{array}{c} \frac{\partial \left(\sum_{i=n-N}^{n}e^{-\frac{{\left(i-n\right)}^{2}}{{2N}^{2}}{\left(D_{i}-{k\times t}_{i}-b\right)}^{2}}\right)}{\partial k}=0\\ \frac{\partial \left(\sum_{i=n-N}^{n}e^{-\frac{{\left(i-n\right)}^{2}}{{2N}^{2}}{\left(D_{i}-{k\times t}_{i}-b\right)}^{2}}\right)}{\partial b}=0 \end{array},$$

Equation (6) is solved to obtain the sum of parameters of the prediction equation, *D* = *k* × *t* + *b*, as expressed by Equation (7),

(7) $$\left[\begin{array}{c} \;k\;\\ \;b\; \end{array}\right]={\left[\begin{array}{cc} \overset{n}{\underset{i=n-N}{\sum }}e^{-\frac{{\left(i-n\right)}^{2}}{{2N}^{2}}}{\times t}_{i}{}^{2} & \overset{n}{\underset{i=n-N}{\sum }}e^{-\frac{{\left(i-n\right)}^{2}}{{2N}^{2}}}{\times t}_{i}\\ \overset{n}{\underset{i=n-N}{\sum }}e^{-\frac{{\left(i-n\right)}^{2}}{{2N}^{2}}}{\times t}_{i} & \overset{n}{\underset{i=n-N}{\sum }}e^{-\frac{{\left(i-n\right)}^{2}}{{2N}^{2}}} \end{array}\right]}^{-1}\cdot \left[\begin{array}{c} \overset{n}{\underset{i=n-N}{\sum }}e^{-\frac{{\left(i-n\right)}^{2}}{{2N}^{2}}}{\times D}_{i}{\times t}_{i}\\ \overset{n}{\underset{i=n-N}{\sum }}e^{-\frac{{\left(i-n\right)}^{2}}{{2N}^{2}}}{\times D}_{i} \end{array}\right],$$

Setting *D* = 0 enables the estimation of the predicted time $t_{0}=-b/k$ of the SUT at the focal position.

Constantly update the prediction value $t_{0}$ according to Algorithm 1. If the current time $t\;\geq \;t_{0}-{\;T}_{1}/2$ and *k* < 0, it is considered that at *t* > $T_{1}/2$, the SUT arrives at the focal point of the microscope’s objective lens. The camera is then triggered to acquire immediately until *t* = $t_{0}{+T}_{1}/2$; at this time the collection of a single-frame image acquisition is completed.

## 4. Fast Spatial Convergence Peak-Extraction Algorithm

At the reconstruction part of the camera, the peak position of the beam’s field intensity distribution has a higher SNR. Commonly used methods to extract the peak position of the spot are the centroid algorithm (CA) [28], Gaussian fitting (GAF) [29], and the circle Hough transform (Hough) [30]. CA has high-operational efficiency, but its peak location accuracy is not high. GAF and Hough are not sensitive to noise but are relatively time-consuming to calculate. Therefore, this study proposes a fast, spatially convergent, peak-extraction algorithm (FSC) that combines both operational efficiency and peak position extraction accuracy. The FSC extracts the peak position of the spot image collected by the area-scan camera. The corresponding relationship between the tilt angle of the SUT and the peak position of the spot image collected by the camera was established.

As shown in Algorithm 2, during the search process for the peak location in a spot image M with an input size m × m using the FSC, the initial search space is placed in the center of the image (m/2, m/2), and the search space radius h is m/2. During the iterative process, the search space is constantly moved and shrunk until the convergence condition is satisfied.
**Algorithm 2.** Fast spatial convergence peak-extraction algorithm**Input:** Area camera acquisition spot image: $M_{m\times m}$
     Iterative convergence threshold: ε
     Minimum spatial scale: $h_{min}$
**Output:** Peak position of $M_{m\times m}$: $\left\{P_{h}{(n),\;P}_{v}\left(n\right)\right\}$
1: Initialize $P_{h}\left(n\right)$←m/2, $P_{h}\left(n\right)$←m/2, n←0, search space radius h←m/2
2: Repeat 3: *n*←*n*+1 4: Create a circle Gauss mask $G_{m\times m}$ with $\left\{P_{h}{(n),\;P}_{v}\left(n\right)\right\}$ as the center and *h* as the radius, and set the mask values in regions beyond the radius *h* to zero 5: $P_{h}\left(n\right)\leftarrow \frac{\sum_{i=1}^{m}\sum_{j=1}^{m}{i\times M}_{m\times m}(i,{j)\times G}_{m\times m}(i,j)}{\sum_{i=1}^{m}\sum_{j=1}^{m}M_{m\times m}(i,{j)\times G}_{m\times m}(i,j)}$, $P_{v}\left(n\right)\leftarrow \frac{\sum_{i=1}^{m}\sum_{j=1}^{m}{j\times M}_{m\times m}(i,{j)\times G}_{m\times m}(i,j)}{\sum_{i=1}^{m}\sum_{j=1}^{m}M_{m\times m}(i,{j)\times G}_{m\times m}(i,j)}$
6: *h*←$h_{\min}+\left(1-\frac{{2h}_{\min}}{m}\right)\times \sqrt{{{(P}_{h}{(n)-P}_{h}(n-1))}^{2}{+(P}_{v}{(n)-P}_{v}(n-{1))}^{2}}$
7: Until convergence: $\sqrt{{{(P}_{h}{(n)-P}_{h}(n-1))}^{2}{+(P}_{v}{(n)-P}_{v}{\left(n-1)\right)}^{2}}\leq \varepsilon$


For the data points in the search space, different weights were assigned according to their distances from the center of the search space. First, as shown in Step 4 of Algorithm 2, a Gaussian mask with a radius h is used to extract the data in the search space. For the data in the search space, the weights are distributed according to a two-dimensional Gaussian distribution with a standard deviation of h according to the distance from the center of the search space. The generated Gaussian mask is given by Equation (8),

(8) $$G_{m\times m}\left(i,j\right)=\{\begin{array}{cc} \frac{1}{\sqrt{2\pi }h}\exp\left(\frac{-\left({\left(i-P_{h}\left(n\right)\right)}^{2}+{\left(j-P_{v}\left(n\right)\right)}^{2}\right)}{{2h}^{2}}\right), & \;{\left({i-P}_{h}\left(n\right)\right)}^{2}+{\left({j-P}_{v}\left(n\right)\right)}^{2}{<h}^{2}\\ \;\;\;\;\;0\;\;\;\;\;\;, & else \end{array},$$

In the iterative process, the center of the search space is updated using step 5 of Algorithm 2, and the center of the search space is closer to the peak location of the light spot. In the initial stage of the iteration, a large search radius h helps improve the search speed of the peak position. In subsequent iterations, a small search radius *h* is helpful for improving the positioning accuracy of the peak position. In step 6 of Algorithm 2, the amount of movement of the search space center in the iterative process is used as the basis for identifying the search process of the peak position so that the algorithm can adaptively adjust the radius of the search space *h* in the iterative process. Moreover, the radius of the search space is limited to the interval between m/2 and the user-set threshold $h_{min}$.

In the calibration experiment, the relationship between the peak position $\left\{P_{h},P_{v}\right\}$ and the tilt angle (*θ*, *φ*) of the SUT is usually established by surface fitting or a surface interpolation algorithm. The polynomial fitting method based on surface fitting is fast; however, the local features of the surface are easily ignored. Biharmonic spline interpolation based on surface interpolation can retain the details of the surface to a great extent [31]; however, the interpolation speed is low and cannot meet the system’s real-time prediction requirements. Therefore, in this study, a partition-fitting polynomial fitting method [15] was used to establish the relation surface between the peak position and (*θ*, *φ*).

## 5. Validation of Simulation Performance

**Figure 4:**
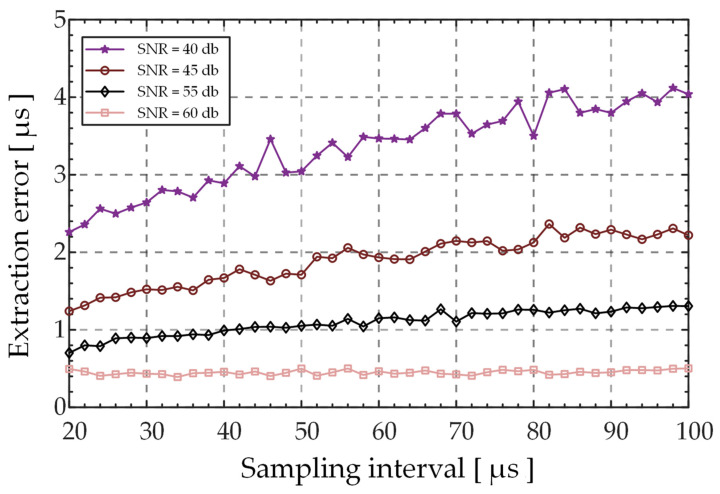
Prediction accuracy at zero-crossing time $t_{0}$ as a function of the sampling interval at different SNR values.

To analyze the influence of the difference signal SNR and sampling interval on the prediction accuracy of the zero-crossing time $t_{0\;}$, and to provide a reference for the selection of sampling intervals in different SNR conditions, this study verified the prediction accuracy of time $t_{0}$ for different differential signals in different SNR conditions and $T_{2}$ sampling intervals based on simulations. The camera exposure time was set to 10 μs, the travel of the differential probe to 100 μm, and the scan frequency to 10 Hz. Equation (4) was used to generate a differential curve, wherein the radius *r* of the Gaussian beam was 3.2 mm, wavelength was 642 nm, the focal length *f* of the microscopic objective was 9 mm, focal length of the imaging lens is 100 mm, pinhole diameter is 10 μm, and pinhole offset is 650 μm. As shown in Figure 4, the average error predicted at zero-crossing time $t_{0}$ was simulated using the zero-crossing prediction algorithm based on the sliding window concept when the sampling interval of the differential signal were in the ranges of 20–100 μs and the SNR of differential signal was within 40–60 dB. During the simulation, the average error for each sampling interval and SNR condition was the result obtained by estimating the mean error of 500 repeated simulations. With an increase in the SNR of the differential signal, the prediction accuracy of the zero-crossing time was significantly improved. For SNR value of 60 dB, with the increase in sampling interval, the prediction error of the zero-crossing time was stable within a certain range. For an SNR value in the range of 40–55 dB, the prediction error tended to increase with the increase in sampling interval, and this trend became more obvious with the decrease in SNR. Thus, improvements in the SNR of the differential signal facilitated higher accuracy of zero-crossing prediction, whereas the reduction in the sampling interval reduced the prediction error of zero-crossing; however, the benefit will be reduced with the increase in SNR.

**Figure 5:**
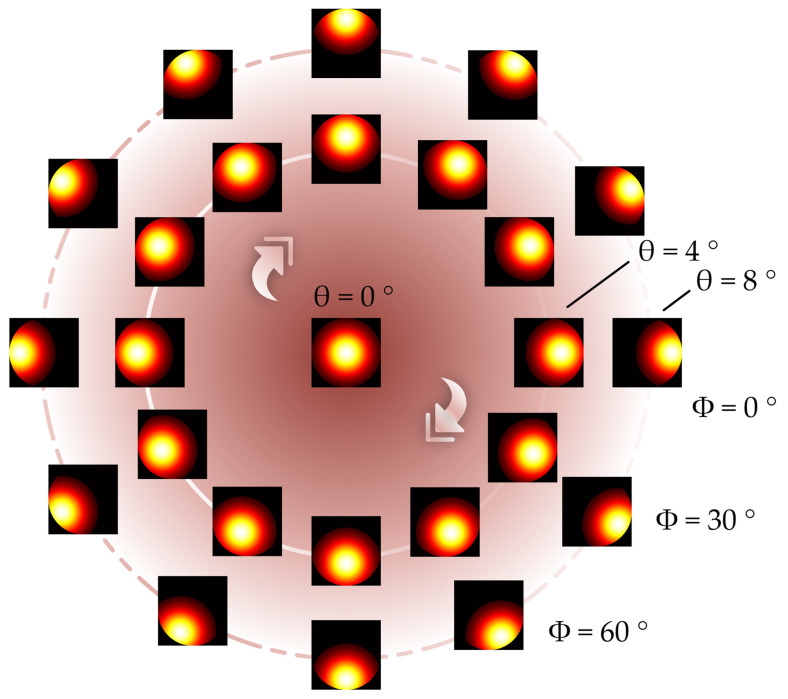
Changes in the peak position of light spot at different tilt angles.

Figure 5 shows the change in the peak position of the spot image received by the camera at different 2D tilt angles in the simulation based on Equation (3). During the simulation, the camera pixel size was 3.76 μm × 3.76 μm, the camera pixel number was 1915 × 1915, the microscope NA was 0.4, the focal length was 9 mm, and the incident beam was a Gaussian with a radius of 3.2 mm. Changes in the tilt angle and direction cause changes in the light spot shape. As the tilt degree *θ* increases, the spot’s peak position offset also increases. However, the tilt direction *φ* only affects the direction in which the peak position of the spot deviates from the center but does not affect its offset.

**Figure 6:**
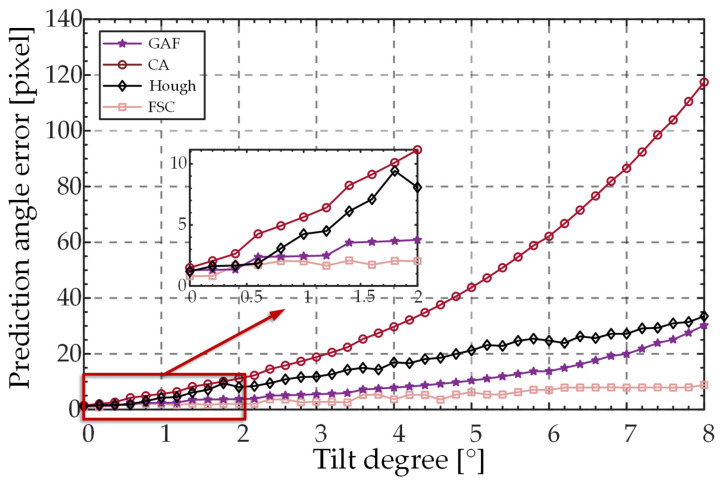
Extraction error of the peak position of light spot by different algorithms.

Figure 6 shows the peak position extraction errors of the different extraction algorithms when the SUT is tilted by *θ* values in the range of 0–8°. As the tilt degree *θ* increases, the asymmetrical degree of the spot shape gradually increases, and the extraction values of CA, GAF, and Hough also increasingly deviate from the peak position, while FSC can still maintain a high peak-extraction accuracy.

## 6. Experiments

**Figure 7:**
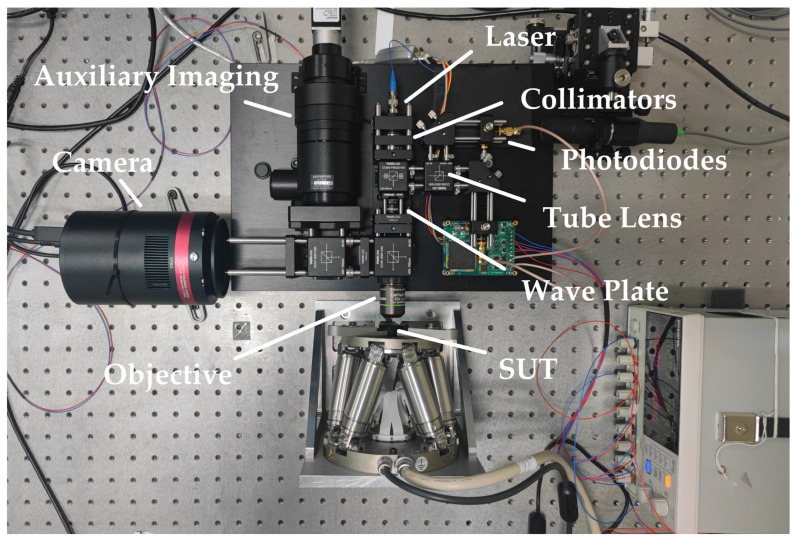
Experimental setup for the measurement system proposed in this study.

The experimental device is shown in Figure 7. At the output of the fiber laser (LP642-PF20, 642 nm, 20 mW, Thorlabs, Newton, NJ, USA), a collimator (F810FC-635, NA = 0.25, *f* = 35.41 mm) was used to collimate the beam. The outgoing beam conformed to a Gaussian distribution with a beam-waist radius of 3.2 mm. The collimated beam passed through a beam splitter, quarter-wave plate (WPQ05ME-633, Ø = 1/2”, Thorlabs, Newton, NJ, USA), and an objective (LMPLFLN 20×, NA = 0.4, *f* = 9 mm, Olympus, Tokyo, Japan), and converged on the surface of the plane mirror (GMH-11, HYGX, Guangzhou, China). The plane mirror was supported by a six-axis displacement platform (H-811. I2, ±10, Power Integrations, San Jose, CA, USA). After the reflected light re-entered the microscope, it was divided into two beams by a beam splitter lens. A beam of light entered a complementary metal-oxide semiconductor camera (QHY600Pro, 9576 × 6388 × 3.76 μm, Light Speed Vision Technology, Beijing, China). The other beam was focused through a flat-convex lens (LA1207-A, Ø1/2”, f = 100.0 mm, Thorlabs, Newton, NJ, USA) and divided into a prefocus and postfocus measurement beam by a beam splitter, and then, respectively, injected pinholes with approximately 650 μm of defocusing. We added an auxiliary imaging device that helped identify the focal plane of the sensor during device tuning. A six-axis displacement table was used with a minimum motion increment of 2.5 μrad and repeatability of ±2 μrad; these technical characteristics met the experimental 2D tilt angle requirements.

When selecting the Gaussian beam radius, the accuracies of differential ranging and tilt angle measurement were weighed. Using a large Gaussian beam radius can improve the efficiency of the numerical aperture and the resolution of the microscope, whereas when using a small radius, the energy of Gaussian beam is more concentrated, which improves the peak-extraction accuracy of the collected spot image. Further, when the SUT is tilted, a part of the beam that deviates excessively from the optical axis cannot return to the pupil plane of the microscope, thus resulting in the reduction in the SNR of the differential signals. Therefore, considering the above factors, we chose a Gaussian beam with a radius of 3.2 mm.

**Figure 8:**
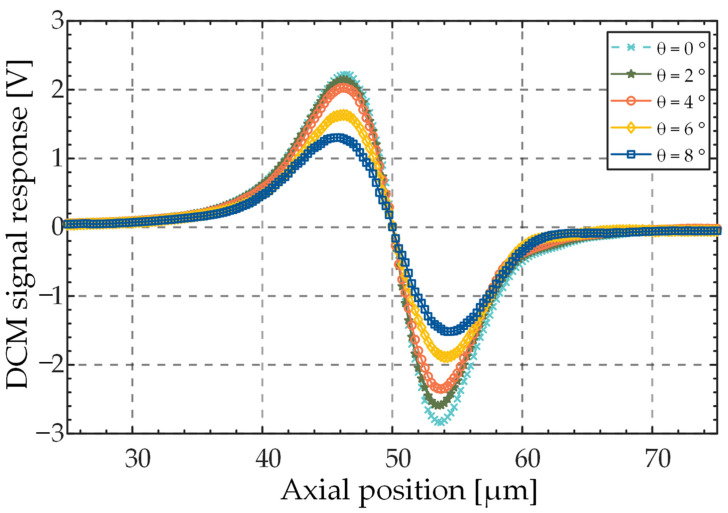
Differential signal curves collected at different tilt degrees.

A slower scanning speed can make the sensor stay near the focal plane for a longer time, thus reserving extended exposure time for the camera, whereas longer camera exposure times can facilitate the capture of spot images with stronger contrast; however, the deviation of exposure position range from zero-crossing results in a reduced ability to predict tilt angle. Thus, considering the use of high-speed real-time scanning in 3D detection, and the fact that the camera only allows a short exposure near the zero-crossing of the differential signal, the exposure time of the camera was set to 40 μs, travel of the differential probe was set to 50 μm, and the axial scanning speed of the six-axis platform was set to 1 mm/s. After the acquisition of a full period of the differential confocal signal, the data in the linear region (the slope was estimated based on the data within the range of 50% of the zero-crossing) were used for linear fitting. The intersection point of the fitted line and time axis was taken as the true value of the zero-crossing time. Figure 8 shows the collected differential signals at different tilt degrees, *θ*. In the range of *θ* from 0° to 8°, 100 sets of differential signal curves were collected for each set of *θ* in increments of 0.1°. The experimental results showed that the average prediction error of the zero-crossing time $t_{0}$ using the zero-crossing prediction algorithm based on the sliding window was 17.63 μs, and the corresponding axial defocus was 17.63 nm.

We verify the effect of zero-crossing prediction error on the peak position of the light spot in the acquired image. The SUT was tilted 8° and placed at a distance of −20 nm, 0 nm, and 20 nm from the focus of the objective lens. 50 light spot images were collected at each position, and the peak position was extracted by the FSC algorithm. The average peak position of spot images extracted from each position was (2512.48 px, 2152.69 px), (2512.62 px, 2152.87 px), (2512.77 px, 2153.04 px). We conclude that the peak position error of the spot image caused by the prediction error of zero-crossing is subpixel level.

**Figure 9:**
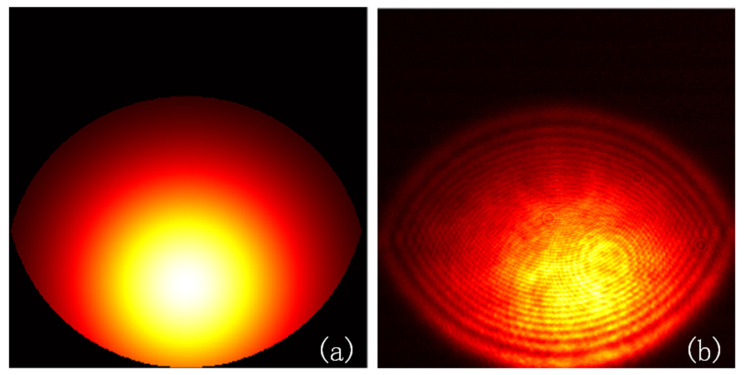
Spot image on the camera when the tilt angle is (6°, 90°). (**a**) Simulated spot image. (**b**) Experimental spot image.

Figure 9 shows a comparison between the simulated and experimental spot images. Because the surface of the SUT was not absolutely smooth, the experimental spot image exhibited a speckle phenomenon. The simulation model only considered the imaging situation of the ideal system; thus, the results of the environmental factors could not be considered. Future studies will address this issue. A comparison of the experimental and simulation results showed that the movement trend of the peak position of the light spot obtained experimentally was consistent with the that related to the simulation results. The simulation model can be used to study the intensity distribution of the reflected light field after tilting the surface and to verify the effectiveness of the peak-extraction algorithm.

In the calibration experiment of the device, the rotation center of the plane mirror was moved to the focal position of the sensor using the six-axis displacement platform such that the plane mirror rotated around the focal position of the sensor and produced different 2D tilt angles; the camera acquired the corresponding spot image. The range of the tilt degree *θ* was 0–8° and values were incremented in 0.1° steps. The range of the tilt direction *φ* was 0–360° and values were incremented in 3° steps. In total, 19,320 training data groups were collected. In addition, 4000 groups of tilt angle data were randomly generated for data collection from the test set.

**Figure 10:**
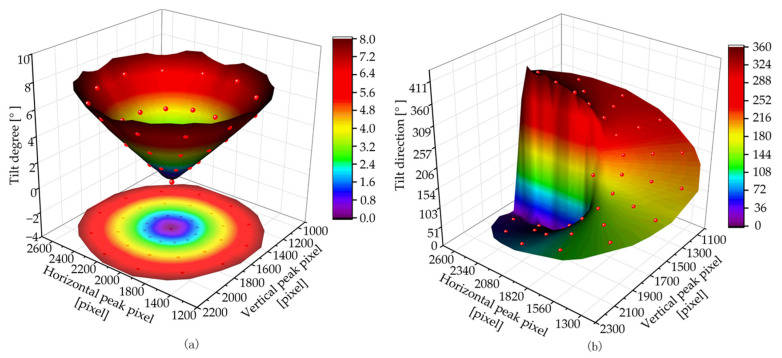
In the equipment calibration experiment, the fitting surface of training set data comprises the relation surface (**a**) between the peak position and *θ*, and the relation surface (**b**) between the peak position and *φ*.

The FSC algorithm was used according to the training set to extract the peak position and establish the relationship between the peak position of the light spot and the tilt angle of the tested surface. Using the partition-fitting polynomial fitting method [15], surfaces determined by the relationship surfaces between the peak position and (a) tilt degree *θ* and (b) tilt direction *φ* were fitted, as shown in Figure 10. 

**Figure 11:**
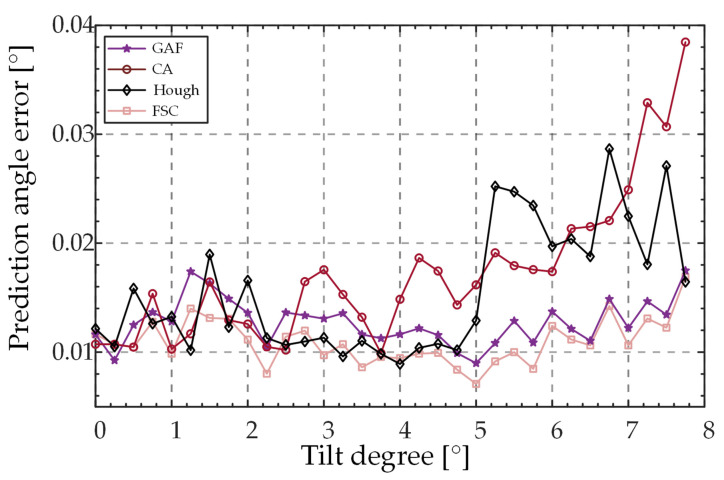
Variation in prediction error *θ* of different algorithms as a function of *θ*.

**Figure 12:**
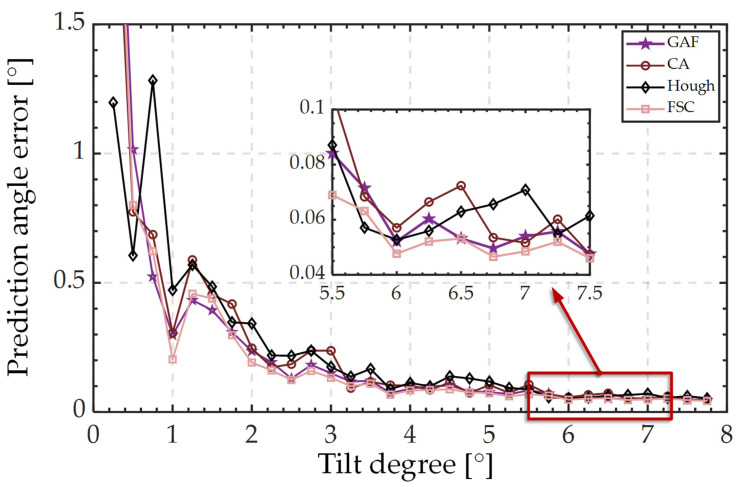
Variation in prediction error *φ* of different algorithms as a function of *θ*.

The measurement accuracy of the equipment was assessed by a test set. Figure 11 shows the change in the prediction error of *θ* as a function of *θ*, and Figure 12 shows the change in the prediction error of *φ* as a function of *θ*. The horizontal coordinate *θ* was separated by 0.2°. Each point in the graph contains the magnitude of the tilt and all the data within a range of +0.2°. The vertical axis represents the average data error within this range. Because the predicted value of *φ* is very unstable when *θ* is less than 0.2°, we only show the prediction error data for *φ* between 0.2° and 8° in Figure 10.

The prediction errors of tilt degree *θ* increase as a function of *θ* when the algorithm CA is used. When using FSC, GAF, and Hough, the prediction error values were in a relatively stable range when *θ* was less than 5°; errors progressively started to show an upward trend when *θ* > 5°. When predicting the tilt direction *φ*, the prediction error values of the four algorithms all exhibited a decreasing trend as a function of *θ*. For the prediction of *θ* and *φ*, the FSC performed better than the other three algorithms.

**Figure 13:**
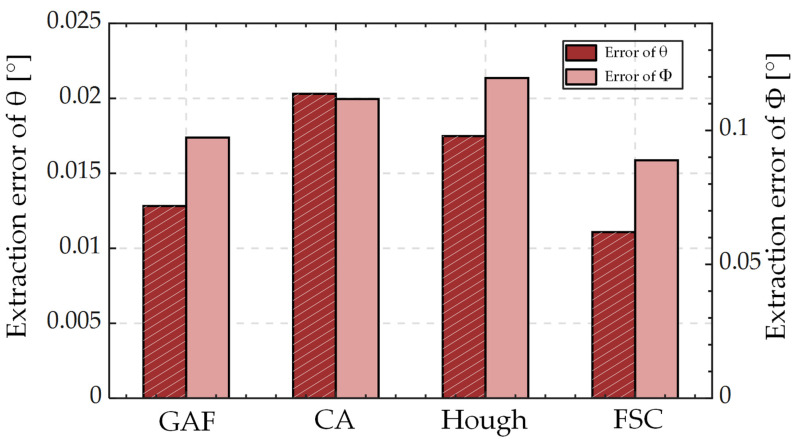
Mean prediction errors of different algorithms.

Figure 13 shows the average angle prediction error using different algorithms in the measurement range  (θ∈[0°, 8°],φ∈[0°, 360°]). FSC, GAF, CA, and Hough were used for spot-image processing. The average prediction errors of *θ* are 0.011°, 0.0123°, 0.020°, and 0.017°, and the standard deviations are 0.010°, 0.016°, 0.029°, and 0.023°, respectively. The average prediction error of *φ* is 0.089°, 0.097°, 0.112°, and 0.120°, and the standard deviations are 0.160°, 0.163° and 0.241°, and 0.200°, respectively. Compared with the other algorithms, the FSC algorithm yielded higher predictive accuracies for *θ* and *φ*.

The proposed technique was implemented in C++ and all the experiments reported in this study were performed on a 2.4 GHz Intel Core 11th Gen PC with 16GB RAM. The processing speed of various algorithms in the measurement range were calculated. The average processing time of FSC, GAF, CA, and Hough were 0.036, 1.372, 0.003, and 0.254 s. For application requirements of scanning speed greater than 10 Hz in 3D detection, the FSC algorithm can consider both the detection accuracy and processing speed.

## 7. Discussion

From the above experiments, we proved the accuracy of the prediction of zero-crossing and the effectiveness of obtaining the tilt angle information of the SUT by using the single image of zero-crossing. Compared with the method of using a cylindrical mirror and linear CCD to obtain the distribution of light intensity on the focal plane of the microscope [15], the structure is simpler and the requirement for the precision of installation is lower. The proposed method can measure the tilt angle of the SUT by collecting images at the zero-crossing of the differential signal. Compared with the method that uses the camera to obtain the diffracted light field distribution of a confocal microscopy system to measure the distance [16], our method can locate the focal plane more accurately. However, the current model of light intensity distribution in the back focal plane does not consider the effects of defocus, aberration, and speckle; hence, it is not able to accurately simulate the change in light spot in the back focal plane. Furthermore, due to the extremely short exposure time of the camera, we can only measure the mirror surface because of the requirements for enhanced camera imaging quality. To meet the measurement requirements of the transmission mirror, the imaging SNR needs to be improved. In addition, owing to the requirement of sensor measurement accuracy, the tilt degree measurement range of this sensor was 0–8°, which can only be applied to the measurement of free-form surface profile with known surface shape and surface shape error within the measurement range.

## 8. Conclusions

This study proposed a sensor that can accurately measure the position and angle simultaneously based on a single acquisition at the zero-crossing, thus enabling the execution of slope measurements by the traditional DCM. First, in the process of measuring the 3D shape of a free-form surface, slope measurements can improve the detection ability of the system for minor defects and can further improve the detection accuracy. Second, the measurement of the slope can be used to obtain the surface shape around the measurement position, which is helpful for the dynamic adjustment of the sampling interval in the detection process. The system uses the excellent prediction ability of the focus position of the differential confocal signal to effectively avoid the influence of the defocus error on the peak migration of the slope measuring unit.

The sensor used the single-exposure camera method at zero-crossing that effectively solved the high-speed, real-time image acquisition problem associated with the optical probe in the traditional DCM due to its long read-out time. A field intensity distribution model of the rear focal plane of the microscope’s objective was established, and the intensity distribution images received by the camera at different 2D tilt angles were simulated. In addition, a zero-crossing prediction algorithm was proposed based on the sliding window concept, and the influences of the sampling interval and SNR on the accuracy of zero-crossing extraction were analyzed. A fast, spatially convergent, peak-extraction algorithm was proposed to solve the problem of peak-extraction accuracy and efficiency. In the equipment calibration experiment, the average error of the zero-crossing prediction of the equipment was 17.63 nm, the average error of the measurement of the tilt degree *θ* in the range of 0–8° was 0.011°, and the average error of the prediction of the tilt direction *φ* in the range of 0–360° was 0.089°. Using this sensor, the spatial position and tilt angle of the surface can be measured accurately.

## Data Availability

Not applicable.
